# Supplementary material for: Characterization of anti-canine CD20 antibody 4E1-7-B_f and comparison with commercially available anti-human CD20 antibodies
Source: PLoS One. 2025 Jun 27;20(6):e0325526. doi: 10.1371/journal.pone.0325526 (PMC12204528; doi:10.1371/journal.pone.0325526)

S1. Fig. Gating strategy for low cytometry analysis

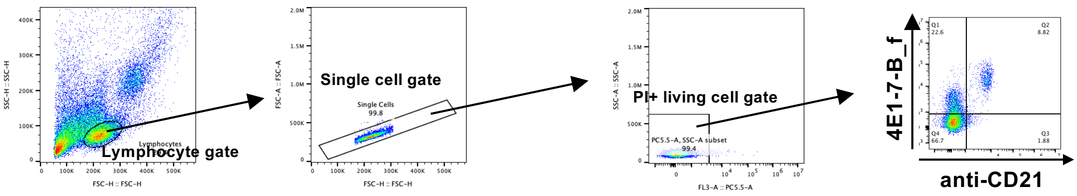

S2 Fig. **Unedited and uncropped full image western blots, Round 1 and Round 2. Round 1 was used in Fig 2 (c)**

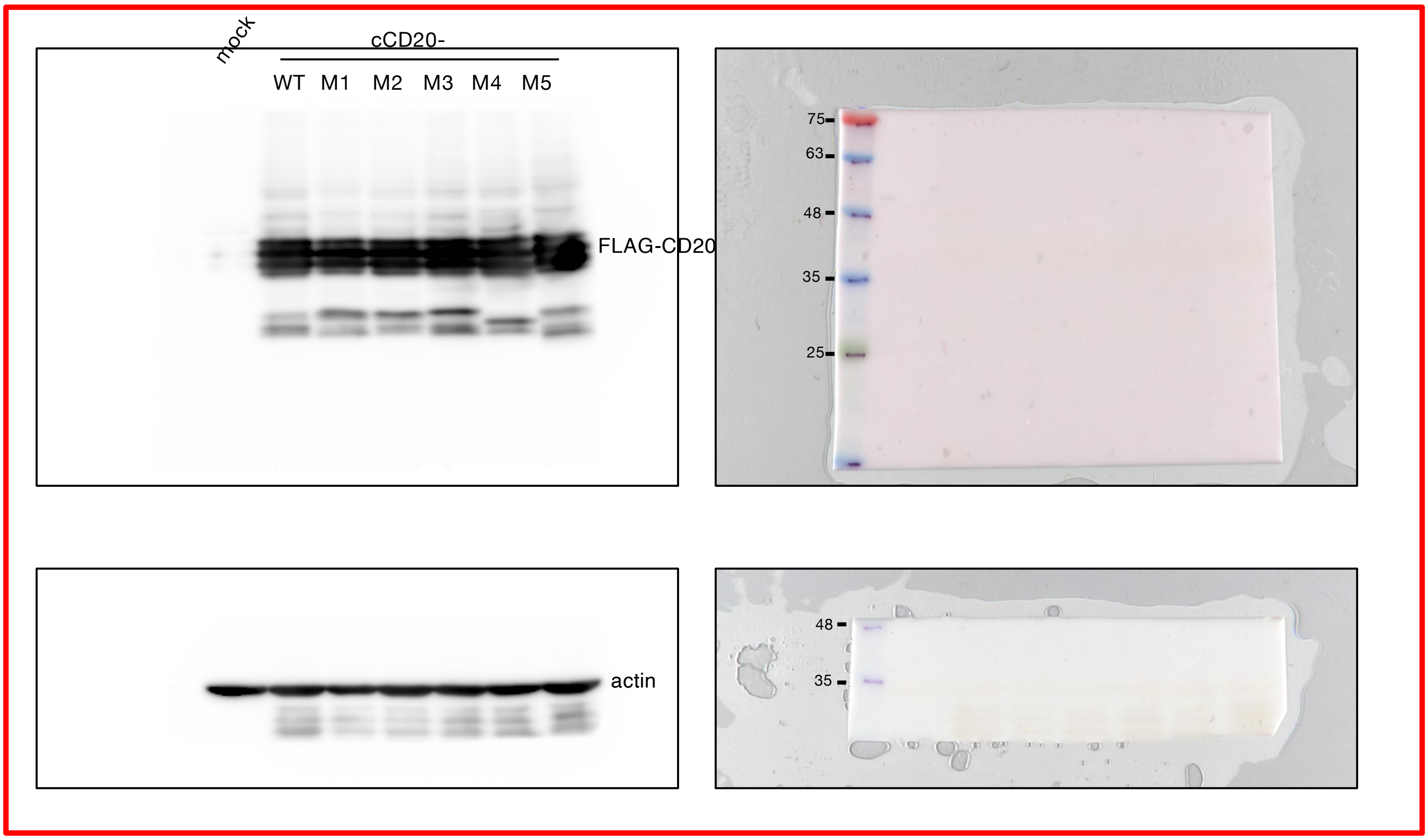

Figure 2(C)

S3 Fig. Unedited and uncropped full image western blots, Round 2.

Loaded samples below are different samples above
obtained from independent experiments .

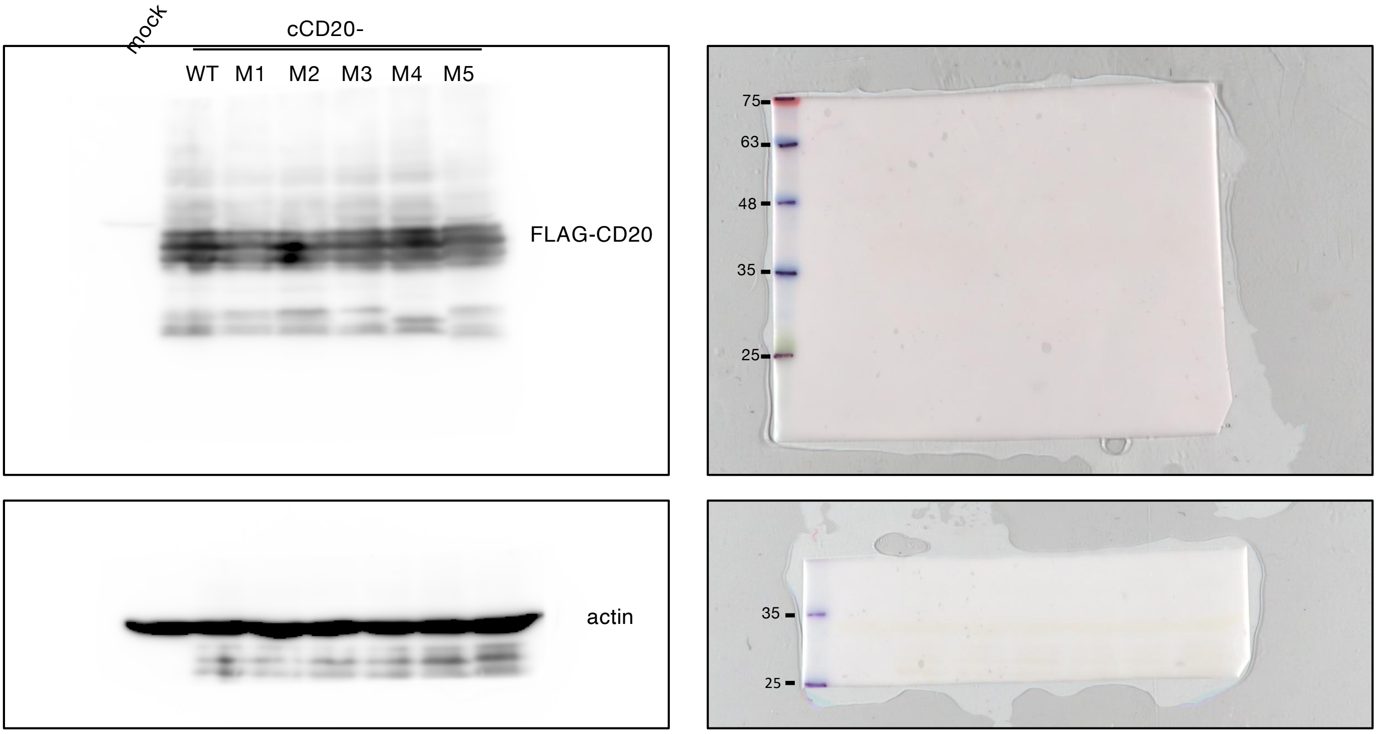

S4 Fig. Unedited and uncropped full image western blots, Round 1 and used in Fig 4.

Figure4

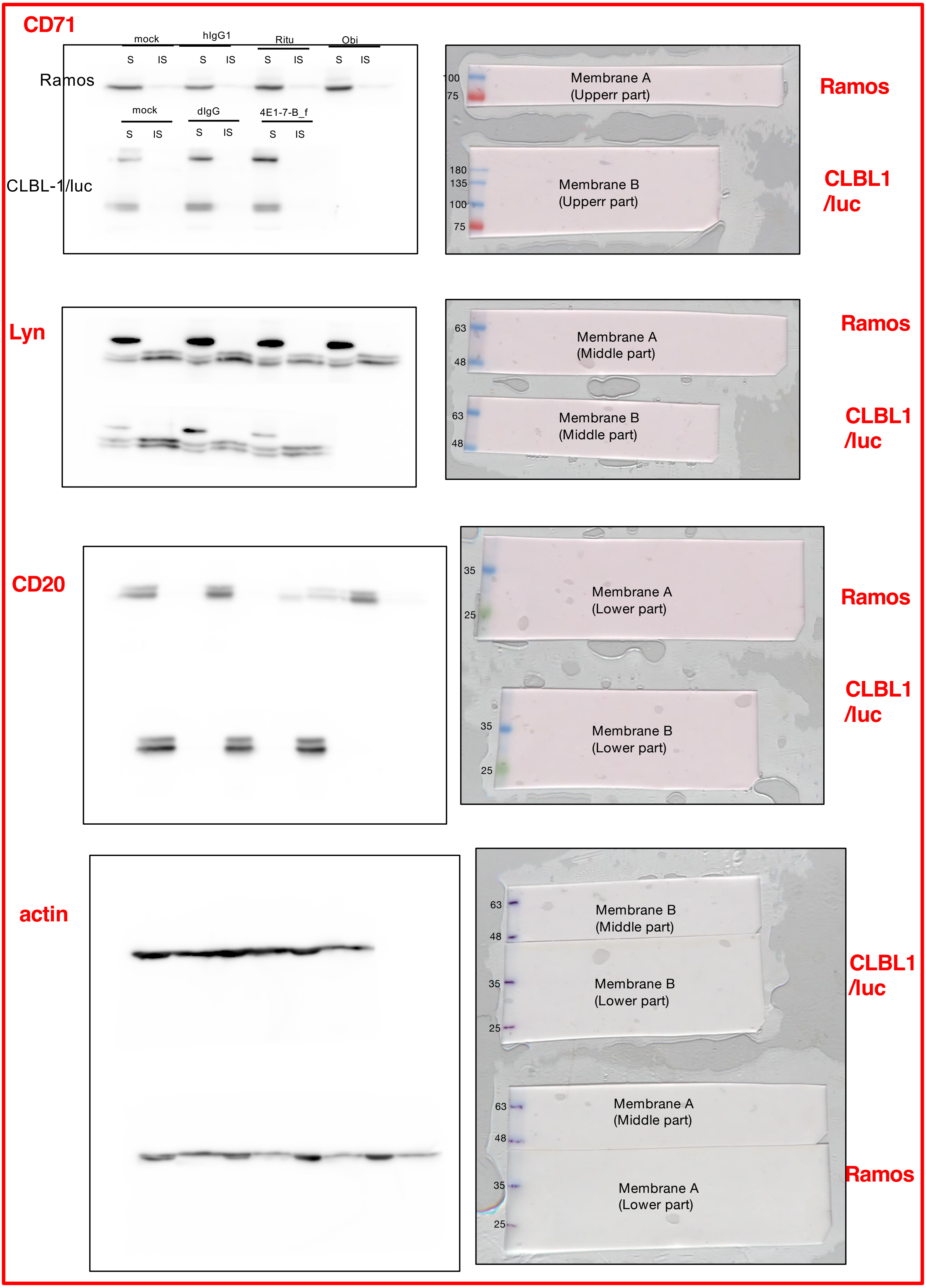

S5 Fig. Unedited and uncropped full image western blots, Round 2.

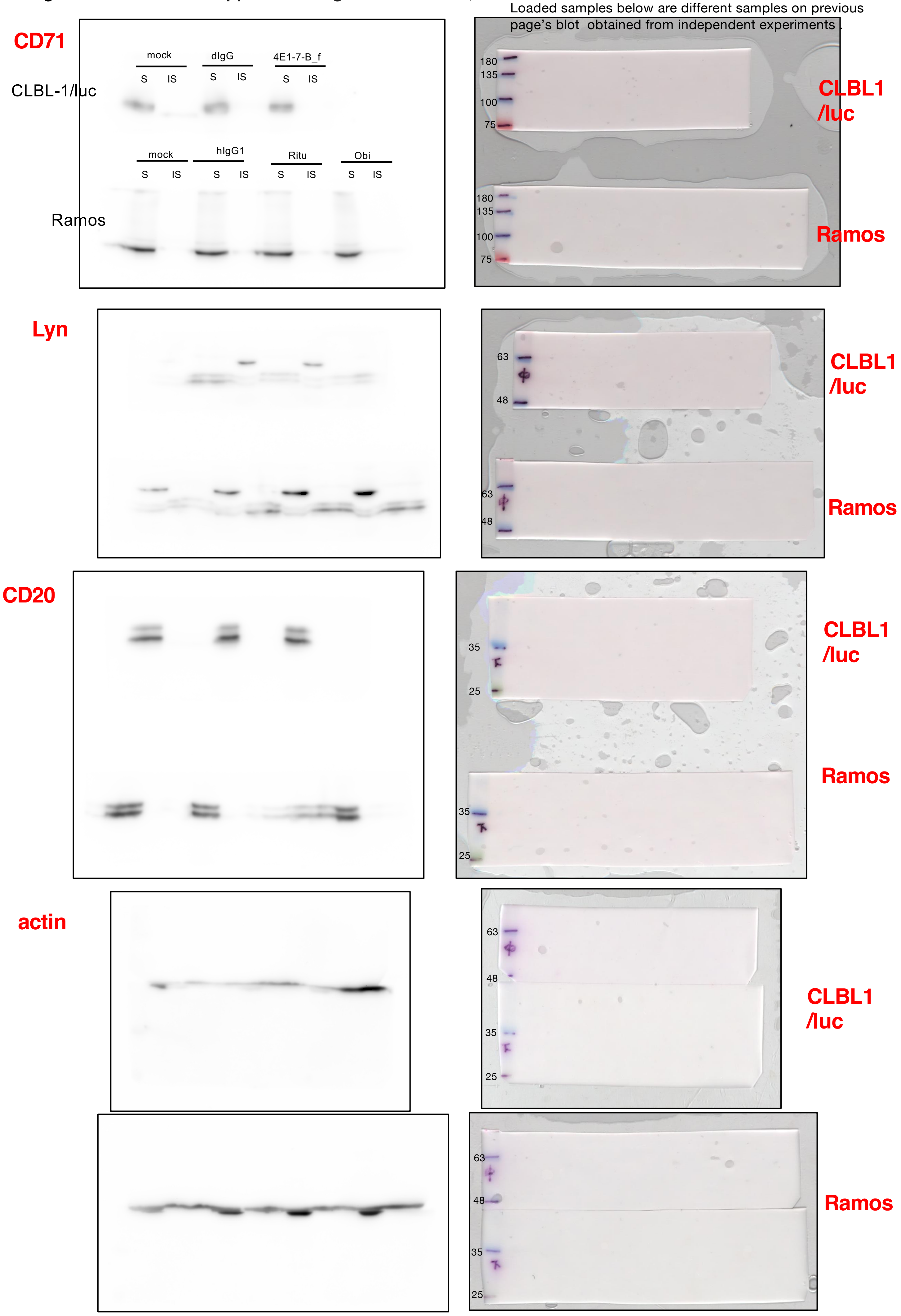

Supplement: S1 File — S1 Fig. Gating strategy for low cytometry analysis. Debris were excluded, and lymphocytes included, using a forward scatter area (FSC) versus side scatter area (SSC) gate. Single cells were then selected on a FSC-A versus FSC-H plot to exclude signaling data from doublets. Dead cells were excluded based on propidium iodide staining and CD21+ and 4E1-7-B_f cells were shown. S2 Fig. Unedited and uncropped full image western blots, Round 1 and used in Fig 2 (c). Full western blot images for the expression of mutant forms of cCD20 (M1 to M5) in NRK cell lines. These westerns are the first of two replicates completed and were used to prepare Fig 2 (c). The order for each lane are: ladder, mock, WT, M1, M2, M3, M4 and M5. S3 Fig. Unedited and uncropped full image western blots, Round 2. Full western blot images for the expression of mutant forms of cCD20 (M1 to M5) in NRK cell lines. These westerns are the second of two replicates completed. The order for each lane are: ladder, mock, WT, M1, M2, M3, M4 and M5. S4 Fig. Unedited and uncropped full image western blots, Round 1 and used in Fig 4. Full western blot images for the expression of CD71, Lyn, CD20 and actin in soluble and insoluble fractions. These westerns are the first of two replicates completed and were used to prepare Fig 4. S5 Fig. Unedited and uncropped full image western blots, Round 2. Full western blot images for the expression of CD71, Lyn, CD20 and actin in soluble and insoluble fractions. These westerns are the second of two replicates completed. (ZIP) [file pone.0325526.s001.zip › Supporting files/S1_Fig.pdf]
