# Supplementary material for: Characterization of anti-canine CD20 antibody 4E1-7-B_f and comparison with commercially available anti-human CD20 antibodies
Source: PLoS One. 2025 Jun 27;20(6):e0325526. doi: 10.1371/journal.pone.0325526 (PMC12204528; doi:10.1371/journal.pone.0325526)
Supplement: Attachment — Submitted filename: 2025.0129 Letter to reviewers.docx [file pone.0325526.s002.docx]

1. Please ensure that your manuscript meets PLOS ONE's style requirements, including those for file naming. The PLOS ONE style templates can be found at
https://journals.plos.org/plosone/s/file?id=wjVg/PLOSOne_formatting_sample_main_body.pdf and
https://journals.plos.org/plosone/s/file?id=ba62/PLOSOne_formatting_sample_title_authors_affiliations.pdf
We have modified it to match PLOS ONE's style requirements as you indicated.

2. Thank you for stating the following financial disclosure:
"T. M. received research funding from Nippon Zenyaku Kogyo Co., Ltd. The remaining authors declare no conflicts of interest."

Please state what role the funders took in the study. If the funders had no role, please state: "The funders had no role in study design, data collection and analysis, decision to publish, or preparation of the manuscript."
If this statement is not correct you must amend it as needed.
Please include this amended Role of Funder statement in your cover letter; we will change the online submission form on your behalf.

Employees of Nippon Zenyaku Kogyo Co., Ltd. are included as co-authors, and their roles are described in the Author contributions.

3. Thank you for stating the following in your Competing Interests section:
"T. M. received research funding from Nippon Zenyaku Kogyo Co., Ltd. The remaining authors declare no conflicts of interest."
Please complete your Competing Interests on the online submission form to state any Competing Interests. If you have no competing interests, please state "The authors have declared that no competing interests exist.", as detailed online in our guide for authors at http://journals.plos.org/plosone/s/submit-now
This information should be included in your cover letter; we will change the online submission form on your behalf.

This information was added into the cover letter.

4. In this instance it seems there may be acceptable restrictions in place that prevent the public sharing of your minimal data. However, in line with our goal of ensuring long-term data availability to all interested researchers, PLOS’ Data Policy states that authors cannot be the sole named individuals responsible for ensuring data access (http://journals.plos.org/plosone/s/data-availability#loc-acceptable-data-sharing-methods).
Data requests to a non-author institutional point of contact, such as a data access or ethics committee, helps guarantee long term stability and availability of data. Providing interested researchers with a durable point of contact ensures data will be accessible even if an author changes email addresses, institutions, or becomes unavailable to answer requests.

Before we proceed with your manuscript, please also provide non-author contact information (phone/email/hyperlink) for a data access committee, ethics committee, or other institutional body to which data requests may be sent. If no institutional body is available to respond to requests for your minimal data, please consider if there any institutional representatives who did not collaborate in the study, and are not listed as authors on the manuscript, who would be able to hold the data and respond to external requests for data access? If so, please provide their contact information (i.e., email address). Please also provide details on how you will ensure persistent or long-term data storage and availability.
We don't have a system to manage such third-party data, so how should we solve this?

5. PLOS ONE now requires that authors provide the original uncropped and unadjusted images underlying all blot or gel results reported in a submission’s figures or Supporting Information files. This policy and the journal’s other requirements for blot/gel reporting and figure preparation are described in detail at https://journals.plos.org/plosone/s/figures#loc-blot-and-gel-reporting-requirements and https://journals.plos.org/plosone/s/figures#loc-preparing-figures-from-image-files. When you submit your revised manuscript, please ensure that your figures adhere fully to these guidelines and provide the original underlying images for all blot or gel data reported in your submission. See the following link for instructions on providing the original image data: https://journals.plos.org/plosone/s/figures#loc-original-images-for-blots-and-gels.
In your cover letter, please note whether your blot/gel image data are in Supporting Information or posted at a public data repository, provide the repository URL if relevant, and provide specific details as to which raw blot/gel images, if any, are not available. Email us at plosone@plos.org if you have any questions.
I attached them in my previous submission.

Reviewers' comments:

Reviewer #1: In the present manuscript, the authors present a sequel on their research on an anti-canine CD20 antibody, which has a significant deleterious effect on CD20-positive cells, showing an ADCC activity and CDC activity (the latter at high concentration of 10 micrograms per mL). Based on its biological activity and the localization to lipid rafts, the authors classify the test antibody as a Type II antibody. In all tests, they compare it with rituximab and glycoengineered obinutuzumab. The experiments they use are well designed and several important controls (isotypes) are used. Certainly, the discovery of novel therapeutic reagents that could combat the wide-spread serious condition of canine lymphoma is a valuable study topic of high translational value.
The delineation of the relevant epitope is also performed, with cell-expressed CD20 mutants that appear of the same expression using a western blot. Interestingly, one of the mutants is irresponsive to the antibody, while few others are less responsive, which is interpreted with a conformational change of the membrane-bound antigen. The expression of the membrane-bound mutant antigen is controlled with an anti-FLAG tag antibody, which might be the reason why the differences in the conformation remained unobserved. The titrations of other anti-CD20 antibodies, such as rituximab, obinutuzumab (or biosimilar), and ofatumumab, as well as the test antibodies could give more insights on the conformational state (and accessibility) of the CD20 mutants used for this study.
Several comments of the authors refer to the antigen affinity of the novel antibody, but at present there are no measurements presented, either titrations or other means of evaluation of the strength of the interaction. This data set should be presented – the manuscript would profit from the exact delineation of the antibody activity in relation to the number of the antigen molecules displayed on the cell surface (especially as the authors use different target cell types to measure the antibody reactivity).
Further, the authors cite only 13 references, which should be expanded, and this should not be difficult for the therapeutically attractive antigen CD20 which is in the centre of this research contribution.
Finally, the conclusion could be oriented into the development of the studied antibody for therapeutic purposes – seeing its activity, probably affinity maturation would be helpful, and these are challenging to design for CD20. Are there data on the expression level, monomeric state, maximal solubility, and stability of the antibody?

To evaluate the affinity of obinutuzumab and 4E1-7-B_f antibodies for NRK/cCD20, we performed a flow cytometry assay using a dilution series of the antibodies and compared the K_D_ values. Similarly, we also performed an assay using CD20 mutants-expressing cells using a dilution series of the 4E1-7-B_f antibody to evaluate their affinity. The data are shown in Figure 2D.

As you indicated, since the number of references cited was small, the revised manuscript cites the newly required references, bringing the total to 21.

We are developing this antibody as a therapeutic animal antibody drug, and in the process, we are studying the expression level, monomeric state, maximum solubility, and stability of the antibody, which are confidential information held by the animal drug company and are difficult to disclose here.

Please find below a list of remarks which I hope you will find helpful.
Line 34: „classified this antibody as a type II antibody similar to the obinutuzumab” – this statement is valuable only to the CD20-specialized audience, I propose to complement it by describing the functional particularities of this antibody class

According to your suggestion, I described the type II antibody in Abstract (Lines 35-37).

Line 46: “Cytotoxic antibody drugs such as anti-CD20 antibodies have antitumor effects through antibody-dependent cell cytotoxicity (ADCC), complement-dependent cytotoxicity (CDC), antibody-dependent cell phagocytosis (ADCP), and direct cytotoxic activity, but these functions vary widely among individual antibody drugs” – this statement should be reworded to point specifically at anti-CD20 antibodies: Anti-CD20 antibodies are cytotoxic antibody drugs exerting antitumor effects…, and concluding in: these functions vary widely among the therapeutic agents directed towards this target, or similar.

According to your suggestion, I modified the sentence (Lines 48-51).

Line 58: their efficacies
Line 62: “no anti-canine CD20 antibody drug is available” – approved? Commercially available?
Line 63: “the functional mechanism“ – mode or mechanism of action
Line 66: based on this molecule.

Line 78: “on the localization of the lipid raft of CD20“ – based on the CD20 localization into lipid rafts?
Line 126: what precisely is FACS buffer composed of?
Line 170: using
Line 177: overexpressed
Line 179: overexpressing

Thank you for your detailed corrections. All have been corrected as you indicated.

Materials and methods: all antibodies should be listed with RRIDs. Isotype controls used for FACS experiments are not mentioned.

I have corrected those as you indicated (Lines 150-199).

Line 206 (and Figure 1C): what is the population reacting with dog isotype control? Were the dead cells gated out?

Yes, the dead cells were gated out by PI staining, which is described in the revised manuscript (Lines 158-159). The population reacting with dog isotype control was Fc-mediated non specific binding of dog IgG isotype control in CD21+ fraction, but we do not know what is the population in CD21- fraction. However, important point is there is no difference between dog IgG binding and 4E1-7-B_f reaction in CD21- faction, but there are increase of binding of 4E1-7-B_f in CD21+ fraction.

Line 259: Measure and extent of statistical significance should be described in the Figure legend.

I have filled in the necessary areas in Figs 2 and 3.

Line 282: “The function of individual cytotoxic antibody drugs is important” – this sentence is very generic, it would be better to reword, that it is crucial to define the mode of action of different cytotoxic antibodies to design their further development, or similar

I have corrected it as you pointed out (Lines 352-353).

Line 298: The comments on affinity, without actual measurements directed in this way, not even titrations of the positive cells, are not well founded at this stage. There are many other factors that can influence the apparent recognition and subsequent binding to the antigen-expressing cells, such as clustering, association with lipid rafts, potential internalization – please consider these in the comments.

The revised manual lists the K_D_ value measured using flow cytometry, but we have also mentioned the possibility you pointed out in lines 374-377.

Line 306: something wrong with the micro (Greek mu) sign

I have corrected.

Paragraph starting with line 301: as mentioned before, it is difficult to judge the affinity based on the reactivity with cell-bound antigens, especially in the case of tetraspanin molecules such as CD20, which show certain patterns of clustering and mobilization in response to antibody treatment.

As mentioned above, explanations have been added where necessary.

Line 311: the reference of Klein et al. is not in the correct format

I have corrected.

Line 322: something wrong with the micro (Greek mu) sign

I have corrected.

Line 327: “is the first antibody that belongs to type II“ – a brief overview of the findings on anti-canine CD20 antibodies would be helpful (although the authors present one in the reference 5).
I have corrected.
