# Supplementary material for: Characterization of anti-canine CD20 antibody 4E1-7-B_f and comparison with commercially available anti-human CD20 antibodies
Source: PLoS One. 2025 Jun 27;20(6):e0325526. doi: 10.1371/journal.pone.0325526 (PMC12204528; doi:10.1371/journal.pone.0325526)
Supplement: Attachment — Submitted filename: 2025.0330 Response to Reviewers.docx [file pone.0325526.s003.docx]

**Dear Editor and Reviewers,**

We would like to thank you for your careful review of our manuscript entitled " Characterization of anti-canine CD20 antibody 4E1-7-B_f and comparison with commercially available anti-human CD20 antibodies" (Manuscript ID: PONE-D-24-19862R1). We sincerely appreciate the insightful and constructive comments provided by the reviewers and the editor, which have helped us to improve the quality and clarity of our work.

We have carefully addressed all the comments and suggestions, and have revised the manuscript accordingly. Below, we provide a detailed, point-by-point response to each of the reviewers’ comments. For clarity, the reviewers’ comments are shown in italic, and our responses are provided below each comment.

We hope that the revised version of the manuscript addresses all concerns and meets the requirements for publication in Plos One.

Sincerely,
Takuya Mizuno (on behalf of all authors)

*Response to Reviewer #1:*

*Line 51: ofatumumab
Line 101: San Diego
Lines 107 and 111: the sequence of the FLAG-tag used here is probably DYKDHDID – this is different from the conventional FLAG sequence, please mention this fact here.
Line 321: this suggests
Line 337: each has a different epitope*
We have corrected those as you indicated.

*Figure 2D: unfortunately, it is not indicated which colour correspond to which dilution, please correct. Additionally, certain mutants are only measured with 3 concentrations of the antibody (3 dots), while others have several? A plot showing dose-response (concentration on x, signal of staining on y axis) should be added here, and EC50 could be added as a figure in the plot or in the text.*

The y-axis of this graph represents the Kd values, and as shown in the M&M they are calculated from the dilution columns of the individual antibodies, so the different colors indicate each Kd value obtained from different independent experiments. The number of dots varies from antibody to antibody because the number of trials for each independent experiment is different. The left plot in Figure 2D has been changed to a plot showing dose-response, as you suggested. Since the Kd values are shown in the graph, which we believe can be evaluated as an alternative to EC50. We have added more details about these in Figure legend (lines 274-279 in the revised manuscript with track changes).

*Epitope mapping experiment is very elegant, but would it be possible to test the mutants for the binding of ofatumumab (M1, 2 and 3) or RX (M4 and 5) and Obinutuzumab (M5) – this could be informative on retaining the CD20 conformation.*

I have conducted the experiment you suggested on rituximab and obinutuzumab. The results are shown in right figure, and we actually found that he M5 mutant was found to be significantly increased in reactivity by both antibodies. These facts, however, are not the subject of our present manuscript and are therefore not included in the text.

*-*

*Response to Reviewer #2: This manuscript is a revised version (I wasn’t involved in the first-round peer-review), overall, they did a great job on responding the reviewer.
I have one minor question:
Fig 3A, isn’t the viability higher in 4E1-7B_f group? It means lower killing of the NK effectors cells, which suggests weak ADCC in the 4E1-7B_f antibody, right? This is the opposite of author’s claim, so please clarify.*

Thank you for your important point, the label on the Y-axis in Figure 3A is incorrect and represents cell injury induced by ADCC, so we have corrected the label on the Y-axis of the graph as such.

*Response to Reviewer #3: Merits：*

*Areas for Improvement:
①Figures and Data Presentation*
*a. Figure 1C: The labeling of CD21+ B cells and gating strategy should be clarified to ensure accurate interpretation.*

We added a description of the gating strategy as Supplement Figure1.

*b. Figure 2D: The statistical significance of binding affinities (KD values) should be explicitly stated in the figure legend.*

As you pointed out, I have explained it in the Figure legend (Lines 270-279 in the revised manuscript with track changes).

*c. Western blot images (Figure 4): The band intensities should be quantified to provide a more objective comparison of lipid raft translocation.*

We have stated that when CLBL-1/luc is treated with 4E1-7-B_f, CD20 does not migrate to the lipid raft. since it is obvious in western blotting that no CD20 band is detected in the IS fraction. We do not believe that reading the intensity would change the results.

*②Clarity in Statistical Methods*
*a. The p-values and statistical test details should be explicitly mentioned for each figure.*

*b. If multiple comparisons were performed, corrections for multiple testing should be addressed.*

We have revised the figure legends to explicitly mention the p-values and details of the statistical tests used for each figure. Additionally, where multiple comparisons were performed, we have addressed corrections for multiple testing accordingly.

*③Expansion of Discussion*
*a, The study should compare 4E1-7-B_f with other reported anti-canine CD20 antibodies beyond rituximab and obinutuzumab.*

There are very limited numbers of published papers about anti-canine CD20 antibodies, but we mentioned about this in lines 338-354 in discussion section of the revised manuscript with track changes.

*b. The discussion should acknowledge potential in vivo challenges, such as immune evasion mechanisms in a clinical setting.*

We mentioned about this in lines 404-415 in discussion section of the revised manuscript with track changes.

*c. The therapeutic implications of a Type II CD20 antibody in long-term B-cell depletion should be elaborated.*

We mentioned about this in lines 395-403 in discussion section of the revised manuscript with track changes.

*④References and Literature Review
The manuscript currently cites only 21 references, which is limited for a study of this scope. Additional literature on anti-CD20 antibody therapy in veterinary oncology should be included.*

We thank the reviewer for this valuable suggestion. In response, we have reviewed the relevant literature more thoroughly and have expanded the reference list to include additional studies related to anti-CD20 antibody therapy. Specifically, we have added references discussing the development and clinical evaluation of anti-canine CD20 antibodies, cross-species reactivity of therapeutic antibodies, and immune evasion mechanisms relevant to antibody therapy. As a result, the number of references has increased from 21 to 28, and the discussion section has been revised accordingly to better place our findings within the context of existing research.

*Response to Reviewer #4:*

*Revision Recommendations
1. Issue: Methods sections lack critical details (e.g., FACS buffer composition, statistical thresholds). Detailed methods ensure reproducibility
Revision:*
*Add precise technical details in Methods (e.g., FACS buffer recipe, criteria for excluding dead cells via PI staining).*

The recipe for the FACS buffer is given on line 137, and the criteria are given on line 149.

*2. Issue: The introduction/discussion underemphasizes the unmet need in canine lymphoma (e.g., chemo resistance rates) and recent advances in veterinary immunotherapy.
Revision:*
*Add statistics on chemo failure rates in canine B-cell lymphoma. Cite 2023–2024 studies on anti-CCR4/Her2 therapies in dogs. Framing 4E1-7-B_f within current veterinary oncology challenges and solutions highlights its translational significance.*

I added line 62-67 regarding “Add statistics on chemo failure rates in canine B-cell lymphoma. Regarding “Cite 2023-2024 studies on anti-CCR4/Her2 therapies in dogs.”, I didn't add it because it has nothing to do with the current study. I also added lines 410-415 and 434-437 in discussion section of the revised manuscript with track changes, regarding “Framing 4E1-7-B_f within current veterinary oncology challenges and solutions highlights its translational significance.”

*3. Issue: Type II antibodies (like obinutuzumab) typically have weak CDC activity, yet 4E1-7-B_f shows moderate CDC at 10 µg/mL. This discrepancy isn’t explained.
Revision:
Explain this discrepancy in the discussion part.*

We have not been able to explain this well: type II antibodies, such as obinutuzumab, have less CDC activity than type I antibodies, but not no CDC activity at all. The fact that our study failed to show CDC activity with obinutuzumab at 10µg/ml and our antibody showed CDC activity does not mean that our antibody has higher CDC activity than obinutuzumab, but rather reflects its affinity for canine CD20. We believe that this is a reflection of the affinity of our antibody for canine CD20. I mentioned this a bit in the discussion section to avoid any misunderstanding by the reader.
